# Management of Onion Thrips (*Thrips tabaci*) in Organic Onion Production Using Multiple IPM Tactics

**DOI:** 10.3390/insects12030207

**Published:** 2021-03-01

**Authors:** Lindsy Iglesias, Michael J. Havey, Brian A. Nault

**Affiliations:** 1 Department of Entomology, Cornell University, Cornell AgriTech, 15 Castle Creek Dr., Geneva, NY 14456, USA; lei7@cornell.edu; 2 Vegetable Crops Research Unit, Department of Horticulture, Agricultural Research Service, U.S. Department of Agriculture, 1575 Linden Drive, University of Wisconsin, Madison, WI 53706, USA; michael.havey@usda.gov

**Keywords:** *Allium cepa* L., spinosad, neem oil, reflective mulch, plant resistance

## Abstract

**Simple Summary:**

Onion thrips (*Thrips tabaci*) is a major pest in organic onion production and effective integrated pest management strategies are lacking. Our objective was to evaluate pest management programs consisting of several different tactics: (1) two onion plant cultivars with semi-glossy leaves (“Rossa di Milano” and B5336AxB5351C) and one with waxy leaves (“Bradley”), (2) silver reflective and white plastic mulches, and (3) with or without an application of a biopesticide (spinosad + neem oil tank mix). Thrips densities were counted weekly and bulbs weighed at harvest. The application of the biopesticide had the most significant reduction in thrips densities and increase in yield. The cultivar “Rossa di Milano” had lower thrips densities compared with “Bradley” and B5336AxB5351C, but also had the lowest yield. Reflective mulch had lower thrips densities than white mulch but had no effect on yield. None of the other tactics provided any significant additional benefits to thrips management. While biopesticides will still be a key component to onion thrips management programs, their application frequency should be further optimized.

**Abstract:**

Onion thrips (*Thrips tabaci* Lindeman) is a major pest in organic onion production and effective integrated pest management strategies are lacking. Our objective was to evaluate combinations of semi-glossy (“Rossa di Milano” and B5336AxB5351C) and waxy (“Bradley”) onion cultivars with reflective mulch, with or without biopesticides (spinosad + neem oil tank mix), to manage *T. tabaci* in organic onion production. Thrips densities were assessed weekly and bulbs graded and weighed at harvest. Onions sprayed with spinosad + neem oil had fewer *T. tabaci* (adults: 74% (2019); larvae: 40% (2018), 84% (2019) and produced higher yields (13% (2018), 23% (2019)) than onions that were unsprayed, regardless of mulch type or onion cultivar. “Rossa di Milano” had relatively fewer adult and larval thrips populations compared with “Bradley” (21% (2018), 32% (2019)) and B5336AxB5351C. However, “Rossa di Milano” had the lowest marketable yield in both years. Reflective mulch reduced densities on certain dates in both years compared to white mulch, but the largest and most consistent reduction only occurred in 2019. Reflective mulch had no impact on bulb yield. While spinosad + neem oil reduced thrips numbers and increased yield alone, none of the treatment combinations were effective at suppressing populations of thrips. Future *T. tabaci* management in organic onions will require optimization of the available effective biopesticides.

## 1. Introduction

*Thrips tabaci* Lindeman (Thysanoptera: Thripidae) is one of the most economically important insect pests of onion (*Allium cepa* L.) worldwide [1,2]. Adults and larvae feed on leaves causing a reduction in photosynthetic production, typically leading to smaller bulb sizes [3]. Feeding injury to onion leaf tissue creates entryways for bacterial and foliar pathogens, which also can be spread among plants by adults [4,5,6,7]. Thrips also vector Iris yellow spot virus (family Bunyaviradae, *Tospovirus* spp. (IYSV)), which can kill onion plants before they fully mature as well as reduce bulb sizes [8,9,10].

Management of *T. tabaci* on onion relies on the use of insecticides in both conventional and organic production systems. Although resistance in *T. tabaci* populations has not been documented to the most commonly used insecticide, spinetoram [11], resistance to pyrethroids and organophosphates has been documented in New York, USA [12,13] and Ontario, Canada [14] and resistance to methomyl and oxamyl (carbamates) and abamectin (Avermectins, Milbemycins) in the Pacific Northwest, USA [15]. Unlike conventional onion production where many insecticides in several different Insecticide Resistance Action Committee (IRAC) classes are available [1,2], organic growers have fewer products allowable by the USDA Standard Organic Practices guidelines [16]. The need for non-chemical strategies for managing *T. tabaci* is crucial to reduce the risk of resistance developing in organic onion production due to repeated use of a limited number of effective active substances. Additionally, non-chemical strategies also can help minimize adverse side effects of pesticides on non-target beneficial organisms, human health, and the environment.

Plastic mulches are used in vegetable production for weed suppression [17,18], soil moisture retention [19,20], reduced soil erosion, modulation of soil temperatures [21,22], and pest management [23,24]. Ultraviolet (UV) reflective mulches have become popular in the production of various crops for their ability to reduce insect pest pressure [25,26,27,28], including thrips. Because thrips locate their host plants in part by using visual cues in the UV spectrum, UV-reflective plastic mulches may obscure host location cues used by thrips [29]. UV reflective silver mulch reduced populations of *Frankliniella occidentalis* (Pergande), *F. tritici*, and *F. bispinosa* in tomato [30] and *F. occidentalis* and *T. palmi* in pepper [31,32]. Less is known about the effect of UV-reflective silver mulches on *T. tabaci* in onion. In two previous studies, silver reflective mulches reduced numbers of *T. tabaci* in onion early in the season with differences fading as the season progressed [33,34].

*Thrips tabaci* has shown affinity toward certain types of onion phenotypes. Phenotypes with blue-gray, “waxy” leaves tend to be more attractive and support higher densities of *T. tabaci* and have more feeding damage compared with those that have lighter-green (“glossy” or “semi-glossy”) leaves [35,36,37,38]. The natural variation of amounts and types of epicuticular waxes on leaves is responsible for these visual differences, and a higher amount of the ketone hentriacontanone-16 (H-16) relative to the other waxes is associated with greater feeding damage [39,40]. Glossy phenotypes have much lower amounts of H-16 compared to waxy onion phenotypes [37]. While low levels of H-16 are beneficial for reducing thrips colonization and their damage, these onion phenotypes tend to be more susceptible to spray damage, foliar pathogens, and excessive transpiration [41]. Onion phenotypes with “semi-glossy” leaves tend to have higher amounts of H-16 than glossy phenotypes, but also higher amounts of other epicuticular waxes. Consequently, some semi-glossy phenotypes may have a similar amount of total waxes as waxy phenotypes, which would provide more protection of the leaves against spray damage and foliar pathogens, but also benefit in being less attractive to and damaged by thrips. Field studies in conventional onion fields in New York have shown that semi-glossy onion cultivars can provide some protection against *T. tabaci*, although increased bacterial disease incidence also occurred [42,43,44]. Similar semi-glossy onion cultivars have not been evaluated in organic production systems.

Organic farms that are certified by the United States Department of Agriculture (USDA) must adhere to strict rules regarding production, food safety, and pest management [16]. Many biopesticides fall within these regulations and must be approved by the Organic Materials Review Institute (OMRI) or the Washington State Department of Agriculture (WSDA) Organic Food Program. Spinosad (Entrust^®^, Corteva Agriscience, Indianapolis, IN, USA), produced from a soil-inhabiting actinomycete bacterium, *Saccharapolyspora spinosa* [45], is one of the most commonly used biopesticides for managing insect pests in organic vegetable production and is effective against several onion insect pests [46,47,48]. Spinetoram (Radiant^®^, Corteva Agriscience, Indianapolis, IN, USA), spinosad’s conventional counterpart, is arguably one of the most effective active ingredients for managing *T. tabaci* in conventional onion production throughout the northern US [1,2,49]. Few other OMRI-listed biopesticides have shown promise for reducing *T. tabaci* densities in onion [48,50,51,52].

An integrated approach for managing *T. tabaci* in onion is crucial due to the lack of available active ingredients that successfully reduce *T. tabaci* populations and the development of insecticide resistance in organic production systems. The objective of our study was to evaluate combinations of UV-reflective mulch and semi-glossy onion cultivars, with and without biopesticides, to determine the most effective integrated management approach for *T. tabaci* in organic onion production. We hypothesized that each management tactic would provide some reduction in thrips densities, but that the most effective strategy would be a combination of UV-reflective mulch, semi-glossy cultivars, and biopesticide applications. We also hypothesized that bulb yield would be greatest following strategies that were most effective against *T. tabaci*.

## 2. Materials and Methods

### 2.1. Experimental Site and Design

Trials were conducted at Cornell University’s AgriTech Gates West Organic Research Farm in Geneva, New York (USA) (42°52′10.2″ N, 77°3′17.47″ W) during the summers of 2018 and 2019. The site was in the process of transitioning to USDA-certified organic land, so virtually all practices followed USDA National Organic Program standards [16]. The land was prepared by disking, millboard plowing, and perfecta cultivation. The soil type was sandy-loam, named “Mineola”. Beds were prepared with 15-0-2 (N-P-K) at 224 kg/ha (Allganic^®^ Nitrogen Plus, SQM Organic North American Corp., Atlanta, GA, USA) plus 5-5-3 (N-P-K) at 504 kg/ha (Earth Friendly^®^, Fertrell, Bainbridge, PA, USA). The beds were fitted with one line of drip irrigation down the center of the beds. Onions (*Allium cepa* L.) were irrigated when necessary to keep the soil moist [53].

Plot beds were 1.4 m wide and separated from adjacent beds by 1.8 m. Plots consisted of two 4.6 m long rows spaced 0.5 m apart and had a total of 90 onions at 0.1 m spacing. Plots within a bed were separated by 0.9 m unplanted buffer. The beds were covered with either white-on-black (1 mm thickness, control) or silver-on-black (1 mm thickness) plastic mulch (Table 1).

The experimental design was a split-split-plot with 12 treatments (2 mulches × 3 cultivars × 2 insecticides) arranged in a randomized complete block design with six replicates (blocks, 72 total plots). Mulch type was considered as the main plot factor, cultivar as the sub-plot factor, and insecticide as the sub-sub-plot factor. Nutrient, water, weed, and disease management followed recommendations for organic onion production in New York [53]. Insecticide treatment applications began when thrips densities reached 0.5 larvae/leaf and continued weekly for seven weeks. Application dates were 7, 12, 19, 26 July and 4, 10, 19 August in 2018 and 3, 10, 18, 25 July and 1, 9, 16 Aug in 2019. Onion thrips larvae were counted weekly for seven weeks, approximately 7 days after each application and before the next application. Details regarding onion thrips sampling are stated below.

When >30% plants in the experimental area had naturally lodged, onions were pulled from the soil and allowed to cure in the field for two weeks prior to harvest. Lodging in onions occurs when plants are nearing final maturity, which is characterized by the cessation of leaf production, softening of the neck, and collapsing of the upright leaves [54]. Details regarding yield data analysis are described below.

### 2.2. Onion Plants

The three onion cultivars evaluated in the study included the waxy cultivar “Bradley” (control), and two semi-glossy cultivars previously shown to suffer less feeding damage by thrips: “Rossa di Milano” and B5336AxB5351C (Table 1). In 2018, all seeds were treated with tetramethylthiuram disulfide (42-S Thiram Fungicide, Bayer CropScience LP, Research Triangle Park, NC, USA) to protect against *Pythium* and *Fusarium* spp. while being propagated in the greenhouse; however, this was determined unnecessary as the level of soil pathogens was low, and seeds were left untreated in 2019. No insecticides were applied to seeds. Onion seeds were planted in potting mix (Pro-Mix PG Organik, Premier Horticulture, Inc., Quakertown, PA, USA) and fertilized via fertigation once per week as a foliar feed with a 2-3-1 nitrogen-phosphorus-potassium (N-P-K) fertilizer at 11.3 mL/L per 10,000 onion transplants (Liquid #3, The Fertrell Company, Bainbridge, PA, USA). Plants were propagated in 96-cell plastic trays in glass greenhouses for 6–8 weeks until they reached the 2- to 3-leaf stage. Greenhouse conditions were 21.1:15.6 °C day:night and 14:10 h light:dark cycle. Plants were then hardened off outdoors for 1–3 weeks before they were transplanted into raised beds on 1 June 2018 and 7 June 2019. Hardening plants is the process of preparing greenhouse-grown seedlings for transplant by exposing them to ambient temperatures and conditions for 1–3 week(s) to minimize transplant plant shock [54].

### 2.3. Pesticides and Application Technique

The OMRI-Listed insecticide treatments included (1) a tank mix of spinosad (Entrust^®^ SC) with neem oil (Trilogy^®^) and (2) unsprayed (“none”, Table 1). The tank mix was chosen because it has been shown to reduced *T. tabaci* populations in organic onions in New York, United States [55]. Pesticides were applied using a CO_2_-pressurized backpack sprayer with a 1.2 m wide boom fitted with four twin-flat fan nozzles (TeeJet-60 8003VS, Spraying Systems, Wheaton, IL, USA) spaced 0.5 m apart and a walking speed of 1.2 m/s calibrated to deliver 337 L/ha at 276 kPa. An OMRI-Listed fungicide copper octoanate (Cueva^®^, Certis USA, LLC, Columbia, MD, USA) was applied at a rate of 1% *v*:*v* to protect the crop from foliar fungal diseases. Fungicides were applied weekly immediately after the insecticide applications and began when signs of foliar disease were observed.

### 2.4. Data Collection

*Thrips tabaci* adult and larval population densities were assessed visually by counting the numbers of each on all leaves from 15 randomly selected onion plants per plot. Thrips were counted from plants within the middle 3.7 m of each plot (i.e., ends of each plot were not sampled). Numbers of onion leaves also were counted weekly to calculate thrips density per leaf. Thrips densities (adults/leaf and larvae/leaf) were calculated by dividing either the total number of adults or larvae in each plot by the number of sampled plants and then by the mean number of leaves per plant in that plot. In New York, *Thrips tabaci* is the dominant species of thrips that infests onion (BAN, personal observation).

Marketable bulb yield was assessed at season end in September in 2018 and 2019. After onions were allowed to dry in the field, all onions from each plot were harvested and leaves trimmed. The bulbs were counted and weighed, and then separated into four grades based on bulb diameter: boiler (<4.8 cm), standard (4.8–7.3 cm), jumbo (7.3–9.5 cm), and colossal (>9.5 cm) [56]. A subsample of 20 of the largest bulbs (jumbo and standard) were cut in half and inspected for bacterial rot diseases. Percent rot was calculated by dividing the number of rotten bulbs by 20 and then multiplying the quotient by 100. Marketable onion yield was calculated by first subtracting the percent rot then dividing the total marketable bulb weight by the total marketable bulb number (=weight/bulb) and finally extrapolating to metric tons/ha based on the planting density and bed distance used in the study. The presence of rot was not different among the treatments.

### 2.5. Data Analysis

All analyses were conducted using a generalized linear mixed model (PROC GLIMMIX, v. 9.4, SAS Institute, Cary, NC, USA) with post hoc analysis conducted where appropriate (*p* ≤ 0.05) using LSMeans with the Tukey Honestly Significant Differences (HSD) adjustment. Data from each year were analyzed separately because environmental conditions were different between years (New York State Integrated Pest Management Program Network for Environment and Weather Applications (NEWA)). All models included mulch, cultivar, mulch × cultivar, insecticide, insecticide × mulch, and insecticide ×cultivar as the fixed effects. The error terms for the split-split-plot design were included as random variables (block × date, block × date × mulch, and block × date × mulch × cultivar). For the repeated measures analysis, date Date was modeled using a repeated measures structure when analyzing thrips densities and was included as a fixed effect in interaction with all the above listed variables.

*Thrips tabaci* adult and larval densities (mean number of adults/leaf and larvae/leaf) were analyzed using models fit to a negative binomial distribution. The mean bulb yield (metric tons/ha) model was fit to a Gaussian distribution and residuals were evaluated for normality. Residuals were found to be normal and did not require a transformation. The proportion of the total bulbs harvested within each size grade was analyzed using a binomial distribution (n bulbs in grade/total bulbs harvested).

## 3. Results

To simplify coverage of these results, only the highest order of significant treatment interactions are included in the main text. All significant lower order interactions and main effects are included in supplemental Figures S1 and S2. Statistics of all effects are included in supplemental Tables S1 and S2.

### 3.1. Impact of Treatments on Adult Thrips tabaci Densities

Densities of adult *T. tabaci* were lower in 2018 than in 2019 (season total mean (± SE) of 0.67 ± 0.02 adults/leaf in 2018 vs. 1.74 ± 0.11 adults/leaf in 2019). Weather conditions were hotter and drier in 2019 than in 2018, which likely contributed to the higher thrips infestation in 2019.

**Figure 1:**
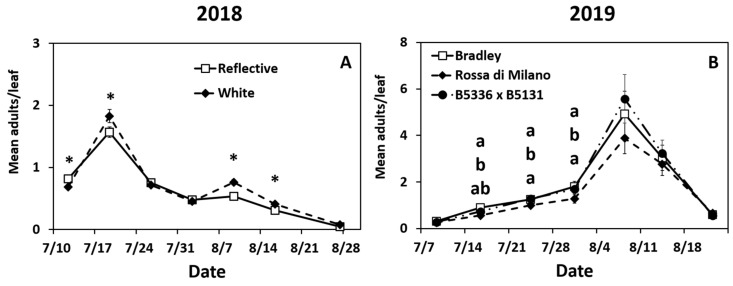
Significant effects of highest order interactions on mean (±SE) adult thrips densities per leaf. Significant effects include the interactions of date × mulch type (**A**) and date × cultivar (**B**) biopesticide in 2018 (**A**) and 2019 (**B**). Thrips were counted weekly for seven weeks. The standard errors at some time points are small and may appear behind the point marker. Means with asterisks (*) or different letters are significantly different at α = 0.05 using Tukey’s LSD post hoc test. Letters are listed from top to bottom in the order of treatments in the legend.

Year 2018: Adult densities peaked during the second week (17 July) and gradually declined until the end of the season (Figure S1A). Adult thrips densities were significantly impacted by the interaction of mulch ×date (Table S1). Adult densities were higher in the reflective mulch on week 1 and higher in the white mulch on weeks 2, 5, and 6 (Figure 1A). Adult densities were also significantly affected by the main effect of mulch (not over time) (Table S1). Onions in the reflective mulch had lower mean season number of adults compared to onions in the white mulch (Figure S1B).

Year 2019: Adult densities gradually increased at the beginning of the season and did not peak until early August (week 5, Figure S1C). The interaction of date × cultivar was significant (Table S1). “Rossa di Milano” had lower densities of adult thrips compared to “Bradley” in weeks 2 through 5 only, and B5336AxB5351C was not different from either cultivar all season (Figure 1B). The interaction of date × mulch × biopesticide was also significant (Table S1). Onions in plots sprayed with spinosad + neem, regardless of mulch type, had lower densities of adult thrips compared to untreated treatments in the first 6 weeks (Table 2). Onions in sprayed plots planted in reflective mulch had lower adult densities than sprayed plots in white mulch for the first five weeks of the season (Table 2).

The interactions of date × mulch and date × biopesticide were also significant (Table S1). Fewer adults per leaf were recorded in the reflective mulch compared to the white mulch in weeks 1 through 4 (Figure S1D). Onions in sprayed plots had lower adult densities than untreated plots throughout the season (Figure S1E). Finally, all three main effects were also significant (Table S1). Fewer adults were recorded in the reflective mulch compared to white mulch (Figure S1F). “Rossa di Milano” had fewer adult thrips than either “Bradley” or B5336AxB5351C (Figure S1G). Onions sprayed with spinosad + neem oil had lower adult densities than untreated onions (Figure S1H).

### 3.2. Impact of Treatments on Larval Thrips tabaci Densities

Densities of larval *T. tabaci* larvae were also lower in 2018 than in 2019 (season total mean (± SE) of 3.62 ± 0.09 larvae/leaf in 2018 vs. 11.9 ± 0.95 larvae/leaf in 2019). As mentioned previously, these results were likely a reflection of hotter and drier weather in 2019.

**Figure 2:**
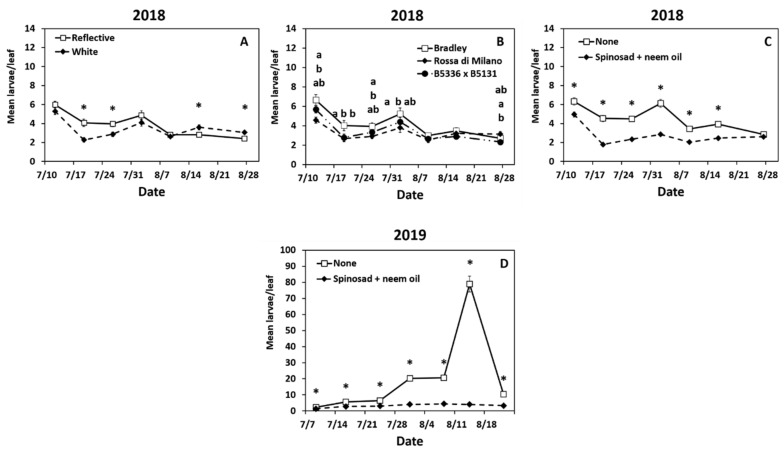
Significant effects of highest order interactions on mean (±SE) larval thrips densities per leaf. Significant effects include the interactions of date × mulch type (**A**), cultivar (**B**), and biopesticide (**C**,**D**) in 2018 (**A**–**C**) and 2019 (**D**). Thrips were counted weekly for seven weeks in Geneva, New York, USA. The standard errors at some time points are small and may appear behind the point marker. Means with asterisks (*) or different letters are significantly different at α = 0.05 using Tukey’s LSD post hoc test. Letters are listed from top to bottom in the order of treatments in the legend.

Year 2018: Larval densities were highest at the start of the season and had a second peak on week 4 (2 August, Figure S2A). Larval thrips densities were significantly impacted by the interactions of date × mulch (Table S1). Adult densities were higher in the reflective mulch on weeks 2 and 3 but higher in the white mulch on weeks 6 and 7 (Figure 2A). The interaction of date × cultivar also significantly impacted larval thrips (Table S1). In the first four weeks, semi-glossy “Rossa di Milano” had lower densities of thrips larvae compared to the susceptible waxy “Bradley” cultivar, whereas semi-glossy cultivar B5336AxB5351C had a lower density than “Bradley” on week 2 only (Figure 2B). Thrips larval densities were significantly affected by the interaction of date × biopesticide (Table S1). The plots sprayed with spinosad + neem had lower larval densities recorded than the untreated plots in the first six weeks of the season (Figure 2C).

The interaction of mulch × biopesticide also was significant (Table S1). Onions sprayed with spinosad + neem oil were lower than untreated onions, regardless of mulch type (Figure S2B). In the untreated onions, lower larval densities were recorded in the white mulch compared to the reflective mulch (Figure S2B). Finally, the main effects of cultivar and biopesticide significantly impacted larval thrips densities (Table S1). The two semi-glossy cultivars “Rossa di Milano” and B5336AxB5351C had lower larval thrips densities compared to the susceptible cultivar “Bradley” (Figure S2C). Spinosad + neem oil reduced densities of thrips larvae compared to the untreated control (Figure S2D).

Year 2019: The densities of onion thrips increased gradually throughout the season and peaked on week 6 (14 August) (Figure S3A). The interaction of date × mulch × cultivar had a significant impact on larval densities (Table S1). Larval densities in “Rossa di Milano” in the reflective mulch were lower than all cultivars in the white mulch early in the season (weeks 1 and 2, Table 3). Onions in the reflective mulch had lower thrips densities than “Bradley” in the white mulch in weeks 2 and 4 (Table 3). The interaction of date × biopesticide was also significant (Table S1). Onions in sprayed plots had lower larval densities than untreated plots throughout the season (Figure 2D).

The interactions of date × mulch and date × cultivar were also significant (Table S1). Fewer larvae per leaf were recorded in the reflective mulch compared to the white mulch in weeks 1 through 4 (Figure S3B). “Rossa di Milano” had lower densities of thrips larvae compared to “Bradley” and B5336AxB5351C across nearly all sampling dates; B5336AxB5351C was not different from Bradley all season except for the last sampling date (Figure S1C).

Finally, all three main effects significantly impacted larval densities (Table S1). The density of larvae in the reflective mulch was lower than the density in the white mulch (Figure S3C), and “Rossa di Milano” had lower densities than both “Bradley” and B5336AxB5351C (Figure S3E). Onions in plots sprayed with spinosad + neem oil had fewer larvae compared with the untreated control (Figure S3F).

### 3.3. Impact of Treatments on Marketable Bulb Yields

Marketable yields (metric tons per ha) were similar in 2018 (mean ± SE = 6.1 ± 0.2) and 2019 (mean ± SE = 7.1 ± 0.2).

**Figure 3:**
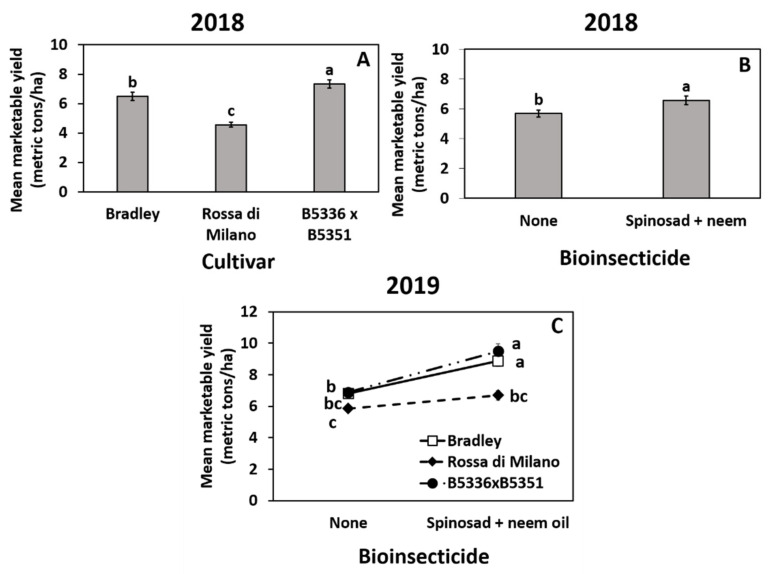
Highest order significant effects of cultivar (**A**), biopesticide (**B**), and the interaction of cultivar ×biopesticide (**C**) on total mean (±SE) marketable onion yield (metric tons per ha) in 2018 (**A**,**B**) and 2019 (**C**) in Geneva, New York, USA. The standard errors at some time points are small and may appear behind the point marker. Means with different letters are significantly different at α = 0.05 using Tukey’s LSD post hoc test.

Year 2018: Mean marketable yield was significantly affected by cultivar and insecticide, but not by mulch type (Table S2). The semi-glossy cultivar B5336AxB5351C had the highest yield, followed by the thrips-susceptible “Bradley” and then semi-glossy “Rossa di Milano” (Figure 3A). Yield was significantly higher in the spinosad + neem oil-treated plots compared to the untreated control (Figure 3B).

Year 2019: The cultivar × insecticide interaction was significant (Table S2). The biopesticide-treated plots planted with “Bradley” and B5336AxB5351C had significantly higher yields than untreated plots regardless of cultivar, whereas total yield in “Rossa di Milano” was similar in both biopesticide-treated and untreated plots (Figure 2C). The main effects of cultivar and insecticide also significantly affected marketable yield, but mulch type did not (Table S2). “Bradley” and B5336AxB5351C both had higher total yield than “Rossa di Milano” (Figure S4A). Similar to 2018, the biopesticide-treated plots had significantly higher yield than the untreated control (Figure S4B).

### 3.4. Impact of Treatments on Marketable Bulb Yield

The proportion of large onion bulbs (diameter > 7.3 cm) in 2018 (mean ± SE = 0.39 ± 0.03) was lower than in 2019 (mean ± SE = 0.61 ± 0.03).

**Figure 4:**
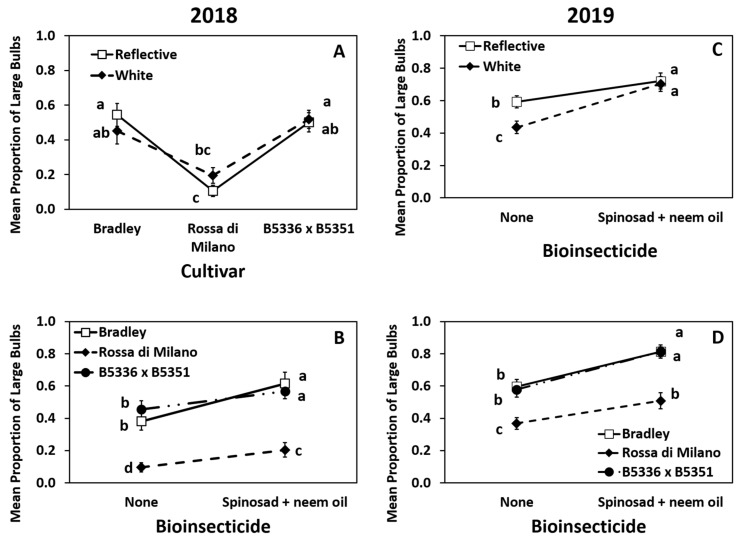
Highest order significant effects of the interactions of mulch × cultivar (**A**), cultivar × biopesticide (**B**,**D**), and mulch by biopesticide (**C**) on mean (±SE) proportion of large onion bulbs (diameter >7.3 cm) in 2018 (**A**,**B**) and 2019 (**C**,**D**) in Geneva, New York, USA. The standard errors at some time points are small and may appear behind the point marker. Means with different letters are significantly different at α = 0.05 using Tukey’s LSD post hoc test.

Year 2018: The interaction of mulch × cultivar was significant (Table S2). The proportion of large bulbs in B5336AxB5351C was similar to the proportion in “Bradley” on both mulch types and significantly greater than the proportion in “Rossa di Milano” on both mulch types (Figure 4A). However, the proportion of large bulbs produced in B5336AxB5351C grown on white mulch was significantly greater than those in “Rossa di Milano” grown on reflective mulch, but not when “Rossa di Milano” was grown on white mulch (Figure 4A). In 2018, the interaction of cultivar × biopesticide was also significant (Table S2). The proportion of large bulbs in all cultivars treated with spinosad + neem oil was greater than those in the same cultivars in untreated plots, but proportions of large bulbs in B5336AxB5351C and “Bradley”, regardless of whether they received a biopesticide, were greater than those in “Rossa di Milano” with or without biopesticide (Figure 4B). The main effects of cultivar and biopesticide were also significant but mulch type was not (Table S3). “Bradley” and B5336AxB5351C had higher proportions of large bulbs compared to “Rossa di Milano” (Figure S4C). Biopesticide-treated plots had significantly higher proportions of large bulbs compared to proportions in the untreated control (Figure S4D).

Year 2019: The interaction of mulch × biopesticide had significant impacts on the proportion of large bulbs (Table S2). Biopesticide-treated plots in both reflective and white mulches had the highest proportion of large bulbs compared to those in untreated plots in both mulch types (Figure 4C). However, the untreated control in reflective mulch had a significantly higher proportion of large bulbs compared to those in the untreated control in white mulch, suggesting that reflective mulch alone had some effect on the production of large bulbs. The cultivar × biopesticide interaction was also significant (Table S2). For all three cultivars, the proportion of large bulbs in plots sprayed with spinosad + neem oil was greater than those in the same cultivars in untreated plots (Figure 4D). The biopesticide-treated “Rossa di Milano” plots had a similar proportion of large bulbs as untreated Bradley and untreated B5336AxB5351C (Figure 4D).

The main effects of cultivar and biopesticide were also significant while mulch type was not (Table S2). Similar to the results in 2018, “Bradley” and B5336AxB5351C had greater proportions of large bulbs than “Rossa di Milano” (Figure S4E), and the insecticide-treated plots had greater proportions of large bulbs than the untreated control (Figure S4F).

## 4. Discussion

Few IPM programs in organic onions include multiple tactics for managing a single insect pest. For example, *T. tabaci* is managed nearly exclusively with insecticides in organic systems. Because insecticides that effectively reduce populations of *T. tabaci* are limited and the potential for insecticide resistance is high in organic production systems, an integrated approach using a combination of non-chemical and chemical strategies is crucial for *T. tabaci* management. Thus, in this study, we examined a multipronged approach to determine the most effective combination of integrated management tactics for *T. tabaci* in onion. We hypothesized that reflective mulch combined with a semi-glossy cultivar and a biopesticide program would maximize onion thrips control. We also hypothesized that bulb yield would be greatest following management strategies that were most effective against *T. tabaci*. While a combination of the semi-glossy cultivar “Rossa di Milano” and the biopesticide treatment spinosad + neem oil significantly reduced thrips densities, reflective mulch had little impact on thrips densities. Despite improving *T. tabaci* management using a combination of “Rossa di Milano” and the biopesticide treatment, total yield and the proportion of large bulbs in this treatment were lower than those in the thrips-susceptible “Bradley” treated with the biopesticide treatment. Similarly, *T. tabaci* densities and marketable yield in the semi-glossy cultivar B5336AxB5351C combined with the biopesticide treatment were comparable to those in the “Bradley” and biopesticide treatment combination. The benefits of reducing *T. tabaci* densities using reflective mulch were slight and inconsistent. However, when reflective mulch was combined with the biopesticide treatment, yields were higher than in the treatment combining white mulch and the biopesticide treatment, regardless of cultivar. Our study highlights the complexity of selecting a combination of IPM tactics that effectively manage *T. tabaci*, while also optimizing yield.

Biopesticide use was the most effective tactic for managing *T. tabaci* in our study. Onions sprayed with spinosad + neem oil had fewer *T. tabaci* and produced higher yields than onions that were unsprayed, regardless of mulch type or onion cultivar. Previous studies in onion production regions of the United States and Pakistan have shown that biopesticides are effective for reducing *T. tabaci* densities in onion [47,57,58,59,60]. However, efficacy depends on the biopesticide applied. Spinosad was the most effective biopesticide for reducing *T. tabaci* densities and improving bulb yield in New York and Wisconsin [46,47,48,55]. Several botanical extracts also have been evaluated for *T. tabaci* control in onions. Azadirachtin, derived from the neem tree seeds or leaves (*Azadirachta indica* Juss), provided moderate reductions in *T. tabaci* densities in studies conducted in the Pakistan [58,60] and feeding damage in studies conducted in New York, United States [48], but improvements in yield were inconsistent. In studies conducted in Ethiopia and Pakistan, Fitiwy et al. [60] and Khaliq et al. [58], respectively, reported reductions in thrips densities of 17.2% and >60% using extracts of *Datura* spp., respectively. Khaliq et al. [58] also found a significant reduction of *T. tabaci* using tree tobacco (*Nicotinia glauca* Graham), as well as a similar yield to the pyrethroid synthetic insecticide lambda-cyhalothrin. Additionally, some of the botanical extracts may be more available and affordable in parts of the world where onions are grown, such as the United States and Canada [60]. Regardless of onion production region, future research should focus on identifying effective rotations of biopesticide products and using action thresholds to reduce potential insecticide resistance.

Leaf phenotype shows some promise as an approach for managing *T. tabaci* in onion. In our study, “Rossa di Milano” had relatively consistent reductions in both adult and larval thrips populations, but this was not evident for B5336AxB5351C. Previous studies indicated that thrips prefer onion cultivars with higher levels of certain epicuticular waxes [1,3,37,39,61]. Higher levels of epicuticular waxes are associated with the thrips-susceptible “Bradley”, whereas “Rossa di Milano” and B5336AxB5351C have characteristically lower wax levels. Cultivars with lower amounts of total wax or H-16 have lower densities of *T. tabaci* and reduced thrips feeding damage [37,38]. The results of our study with “Rossa di Milano” were consistent with these findings. However, the level of thrips damage on cultivars with unique wax profiles can vary based on the number of leaves, time to maturity, and leaf structure (open vs. closed neck), as well as amounts of waxes [3,62], which could explain the disparate results we found between “Rossa di Milano” and B5336AxB5351C.

Semi-glossy cultivars must have additional properties that make them acceptable replacements for thrips-susceptible cultivars. “Rossa di Milano” consistently had lower thrips densities, but also had lower marketable total yield than “Bradley”. One potential reason for the lower yield in “Rossa di Milano” is that it simply has a lower yield potential than “Bradley” and such a comparison is not meaningful. The replacement of “Bradley” with a lower yielding thrips-resistant cultivar is not likely to be adopted by onion growers unless fewer insecticide applications are required to protect it against *T. tabaci*. Ideally, resistant cultivars should reduce onion thrips densities below action thresholds, thereby reducing the number of insecticide applications applied throughout the season [42,49]. In our studies, the mean season densities of onion thrips larvae in the semi-glossy cultivars were above 3 larvae/leaf in 2018 and above 10 larvae/leaf in 2019. An economic injury level (EIL) has been reported as a season mean of 2.2 thrips/leaf in conventionally produced onion [63,64], but no EILs have been generated for organic onions. If using a similar EIL, the semi-glossy cultivars in our study would not have been effective enough to withstand *T. tabaci* infestations without the use of biopesticides.

Reflective mulch was not a highly effective cultural management tactic for *T. tabaci* in our study. Reflective mulch showed an inconsistent reduction of adults and larval populations on certain sampling dates throughout both seasons. This result is in contrast to our hypothesis that reflective mulch would significantly delay thrips infestations by reducing thrips colonization early in the season. Because silver reflective mulch works by reflecting shortwave ultraviolet (UV) wavelengths of sunlight, which confuses and repels incoming adults [29], we expected that the effect of the reflective mulch would be most prominent in the early season before the onion plant canopy grew large enough to minimize the reflection. We observed reductions in thrips densities on certain dates in both years, but the largest and most consistent reduction in the early season was only seen in 2019, when adult and larval thrips densities were 2–3 times lower than in the white mulch, suggesting that the benefits of reflective mulch may be most evident during hot dry years when *T. tabaci* pressure is highest. Although reflective mulches have been used successfully to reduce other insect pest densities including thrips [25,28,31], research on *T. tabaci* has been limited and the results are inconsistent. While van Toor et al. [34] saw no significant differences in *T. tabaci* densities among UV-reflective or silver reflective mulches compared with bare ground, Lu et al. [33] found that reflective mulches were effective at reducing numbers of adult *T. tabaci* when shallots were spaced further apart. This result is consistent with the hypothesis that as the plant canopy shades the reflective mulch, the mulch becomes less effective. Even when combined with other IPM tactics, reflective mulch had little effect on reducing *T. tabaci* densities in our study.

Reflective mulches provide other benefits to vegetable growers. Silver mulches have improved yields of squash [25], peppers [28], and tomatoes [30]. Hoepting et al. [65] reported an increase in marketable onion yield that was almost three times greater than that of marketable yield from onions grown on black plastic mulch. In our study, reflective mulch did not improve marketable yield or the proportion of large bulbs compared with those grown on white mulch. Reflective mulches can also reduce pathogen incidence. Hoepting et al. [65] found that silver mulches reduced bacterial bulb rots 59–75% in onions compared with those grown on bare ground in New York and Pennsylvania. Nyoike et al. [26] saw a reduction in Cucumber Leaf Crumple Virus (CuLCrV) transmitted by the silverleaf whitefly (*Bemesia tabaci* (Gennadius)) in zucchini squash with the use of silver reflective mulch compared to bare ground. Incidence of Tomato Spotted Wilt Virus (TSWV) vectored by *Frankliniella* spp. was reduced in tomatoes planted in reflective mulch compared to black mulch [30]. Although reflective mulches may not consistently reduce *T. tabaci* infestations or improve onion yield in New York, there is a possibility that they may reduce Iris Yellow Spot Virus (IYSV), which is transmitted by *T. tabaci* and can be an economically devastating problem in many onion-producing regions in the United States [10,66].

As far as identifying an optimal combination of IPM tactics for managing *T. tabaci* in organic onion production, the results from our study failed to show consistent and significant benefits of non-chemical tactics. The application of a tank mix of spinosad + neem oil was highly effective and consistent for *T. tabaci* management. Further research on relevant economic injury levels for *T. tabaci* in organic onion production is a necessary first step for measuring the effect of some of the evaluated cultural strategies. For example, the small reduction we saw in thrips numbers in onion grown in reflective mulch may not have translated to improved yield, but any reduction in the number of biopesticide applications during the season is unknown. Additionally, other effective biopesticides are needed for *T. tabaci* to prevent insecticide resistance in these systems. Action thresholds for spinosad + neem oil are also needed to optimize use of spinosad. Finally, similar research should be conducted in other regions where benefits of multiple IPM tactics, especially non-chemical tactics, may occur.

## 5. Conclusions

While *T. tabaci* pest management in all onion systems is complex, pest management in organic systems proves to be more restrictive and challenging. Our study indicates that biopesticides continue to be the most consistently effective tactic for managing *T. tabaci* in organic onions. Although this result is promising, the availability of only one effective tactic can be problematic. *Thrips tabaci* is susceptible to developing insecticide resistance and reduced effectiveness of conventional insecticides has been reported [12,13,14,15]. As a result, optimization of insecticide applications in the form of action thresholds, economic injury levels, and rotation of chemical classes has been implemented in parts of the United States [43,44,46,49,63]. The future of organic management of *T. tabaci* would benefit from these same strategies and could sustain the effectiveness of the few products available to organic growers.

## Figures and Tables

**Table 1:** Treatments evaluated in onion trials in 2018 and 2019.

Treatment	Level	Manufacturer	Rate
Mulch	White	FilmTech Corp., Allentown, PA	-
	Silver	Rain-Flo Irrigation, East Earl, PA	-
Cultivar	“Bradley”	Waxy hybrid from Bejo Seeds, Geneva, NY	-
	“Rossa di Milano”	Open pollinated population with semi-glossy foliage from Johnny’s Selected Seeds, Winslow, ME	-
	B5336AxB5351C	Experimental hybrid with semi-glossy foliage developed by MJH	-
Pesticide	Spinosad + neem oil (tank mix)	Corteva Agriscience, Indianapolis, IN + Certis USA, LLC, Columbia, MD	0.6 L/ha + 1% *v*:*v*
	None	-	-

**Table 2:** Significant effects of highest order interactions on mean (±SE) adult thrips densities per leaf. Significant effects include the interactions of date × mulch type × biopesticide in 2019. Thrips were counted weekly for seven weeks. Means with different letters within a row are significantly different at α = 0.05 using Tukey’s LSD post hoc test.

		Reflective Mulch (Mean ± SE)		White Mulch (Mean ± SE)	
Date	Biopesticide	None	Spinosad + Neem Oil	None	Spinosad + Neem Oil
07/09/19		0.19 ± 0.01a	0.17 ± 0.02b	0.43 ± 0.02a	0.33 ± 0.02b
07/16/19		0.62 ± 0.06a	0.45 ± 0.04b	1.07 ± 0.09a	0.75 ± 0.06c
07/24/19		1.12 ± 0.09a	0.69 ± 0.06b	1.80 ± 0.11b	1.08 ± 0.08c
07/31/19		1.79 ± 0.16a	0.68 ± 0.05b	2.93 ± 0.18c	0.95 ± 0.08d
08/08/19		7.83 ± 0.85a	0.80 ± 0.07b	9.30 ± 0.65c	1.24 ± 0.09d
08/14/19		5.91 ± 0.39b	0.59 ± 0.06a	4.70 ± 0.35c	0.82 ± 0.07c
08/22/19		0.61 ± 0.06a	0.65 ± 0.08ab	0.47 ± 0.03b	0.70 ± 0.07ab

**Table 3:** Significant effects of highest order interactions on mean (±SE) adult thrips densities per leaf. Significant effects include the interactions of date × mulch type × biopesticide in 2019. Thrips were counted weekly for seven weeks. Means with different letters within a row are significantly different at α = 0.05 using Tukey’s LSD post hoc test.

		Reflective (Mean ±SE)			White (Mean ±SE)		
Date	Cultivar	Bradley	Rossa	B5336xB5351	Bradley	Rossa	B5336xB5351
07/09/19		0.22 ± 0.02c	0.15 ± 0.01c	0.18 ± 0.02bc	0.40 ± 0.03a	0.37 ± 0.02ab	0.37 ± 0.03ab
07/16/19		0.68 ± 0.06b	0.40 ± 0.05c	0.53 ± 0.08bc	1.10 ± 0.10a	0.72 ± 0.06ab	0.91 ± 0.11ab
07/24/19		1.00 ± 0.08a	0.71 ± 0.06b	0.99 ± 0.15ab	1.49 ± 0.13a	1.27 ± 0.16ab	1.56 ± 0.18a
07/31/19		1.49 ± 0.25bc	0.89 ± 0.13d	1.33 ± 0.22bc	2.10 ± 0.39a	1.68 ± 0.33cd	2.04 ± 0.29ab
08/08/19		4.87 ± 1.48ab	3.17 ± 0.79c	4.89 ± 1.44ab	4.98 ± 1.33a	4.57 ± 1.05bc	6.26 ± 1.55ab
08/14/19		3.58 ± 0.94a	2.68 ± 0.72b	3.49 ± 0.92a	2.49 ± 0.53ab	2.85 ± 0.70a	2.95 ± 0.74ab
08/22/19		0.64 ± 0.05	0.70 ± 0.11	0.53 ± 0.10	0.54 ± 0.07	0.62 ± 0.08	0.59 ± 0.07

## Supplementary Materials

The following are available online at https://www.mdpi.com/2075-4450/12/3/207/s1, Table S1: Statistics for mean adult and larval thrips densities, Table S2: Statistics for bulb yields, Figure S1: Significant lower order effects on adult thrips densities, Figure S2: Significant lower order effects on larval thrips densities in 2018, Figure S3: Significant lower order effects on larval thrips densities in 2019, Figure S4: Significant lower order effects on bulb yield.
