# Supplementary material for: Management of Onion Thrips (Thrips tabaci) in Organic Onion Production Using Multiple IPM Tactics
Source: Insects. 2021 Mar 1;12(3):207. doi: 10.3390/insects12030207 (PMC8000123; doi:10.3390/insects12030207)
Supplement: Supplementary file 1 [file insects-12-00207-s001.pdf]

Supplemental material

Table S1. Statistics (*F*, DF, *P*) for mean adult and larval thrips density repeated measures analyses using PROC GLIMMIX (SAS, Cary, NC).

		Adult Thrips (*F*, *P*)		Larval Thrips (*F*, *P*)	
Effect	DF	2018	2019	2018	2019
date	6, 404	**336.7, <0.0001**	**277.76, <0.0001**	**61.58, <0.0001**	**181.97, <0.0001**
mulch	1, 8	**5.91, 0.0412**	**62.11, <0.0001**	3.82, 0.0863	**33.49, 0.0004**
mulch*date	6, 404	**8.62, <0.0001**	**8.8, <0.0001**	**12.76, <0.0001**	**9.28, <0.0001**
cult	2, 18	0.76, 0.4825	**10.42, 0.001**	**9.19, 0.0018**	**13.46, 0.0003**
mulch*cult	2, 18	0.08, 0.9267	2.81, 0.087	0.38, 0.6881	3.16, 0.0669
cult*date	12, 404	1.32, 0.2031	**2.14, 0.014**	**2.89, 0.0007**	**4.46, <0.0001**
mulch*cult*date	12, 404	0.71, 0.7402	0.31, 0.9884	0.8, 0.6459	**2.06, 0.0186**
insecticide	1, 404	2.63, 0.1055	**632.34, <0.0001**	**275.51, <0.0001**	**781.2, <0.0001**
mulch*insecticide	1, 404	0.29, 0.5879	2.74, 0.0987	**3.89, 0.0492**	3.77, 0.0527
cult*insecticide	2, 404	0.87, 0.4211	1.49, 0.2275	0.5, 0.6071	1.51, 0.222
insecticide*date	6, 404	1.61, 0.1436	**123.5, <0.0001**	**14.59, <0.0001**	**85.87, <0.0001**
mulch*insecticide* date	6, 404	1.14, 0.3397	**2.77, 0.0118**	1.17, 0.3215	0.6, 0.7333
cult*insecticide* date	12, 404	0.38, 0.9696	1.01, 0.4359	0.5, 0.9132	1.56, 0.1022

[1] Values in bold are significant at α=0.05.

Table S2. Statistics (*F*, DF, *P*) for proportion of large onion bulbs (diameter >7.3 cm) and mean total yield (metric tons/ha) analyses using PROC GLIMMIX (SAS, Cary, NC).

		Marketable Yield (metric tons/ha) (*F*, *P*)		Proportion Large Bulbs (*F*, *P*)	
Effect	DF	2018	2019	2018	2019
mulch	1, 5	0.09, 0.7766	1.01, 0.3602	0.04, 0.8518	4.70, 0.0824
cult	2, 20	**97.68, <0.0001**	**27.41, <0.0001**	**68.45, <0.0001**	**35.79, <0.0001**
mulch*cult	2, 20	3.30, 0.0578	0.17, 0.8486	**4.80, 0.0198**	0.06, 0.9404
insecticide	1, 32	**32.69, <0.0001**	**148.02, <0.0001**	**116.87, <0.0001**	**288.55, <0.0001**
cult*insecticide	2, 32	2.52, 0.0965	**17.18, <0.0001**	**9.71, 0.0005**	**10.45, 0.0003**
mulch*insecticide	1, 32	1.09, 0.3049	0.99, 0.3273	0.26, 0.6164	**26.89, <0.0001**

[1] Values in bold are significant at α=0.05.

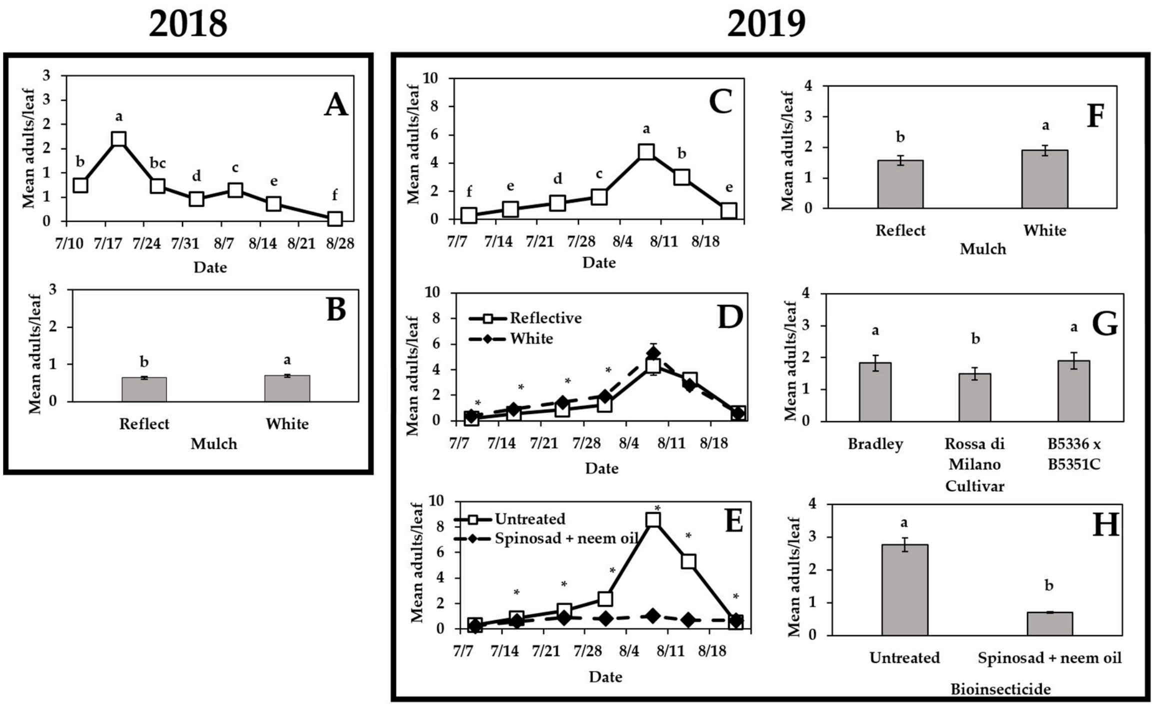

Figure S1. Significant lower order effects of interactions on adult thrips densities not included in the main text. Effects of date (A, C), mulch (B, F), cultivar (G), bioinsecticide (H), and the interactions of mulch by date (D), and bioinsecticide by date (E) on mean (±SE) adult thrips densities in Geneva, New York, USA. Thrips densities are calculated as thrips/leaf. Means with asterisks (*) or different letters are significantly different at α = 0.05 using Tukey's LSD posthoc test. Letters are listed from top to bottom in the order of treatments in the legend.

2018

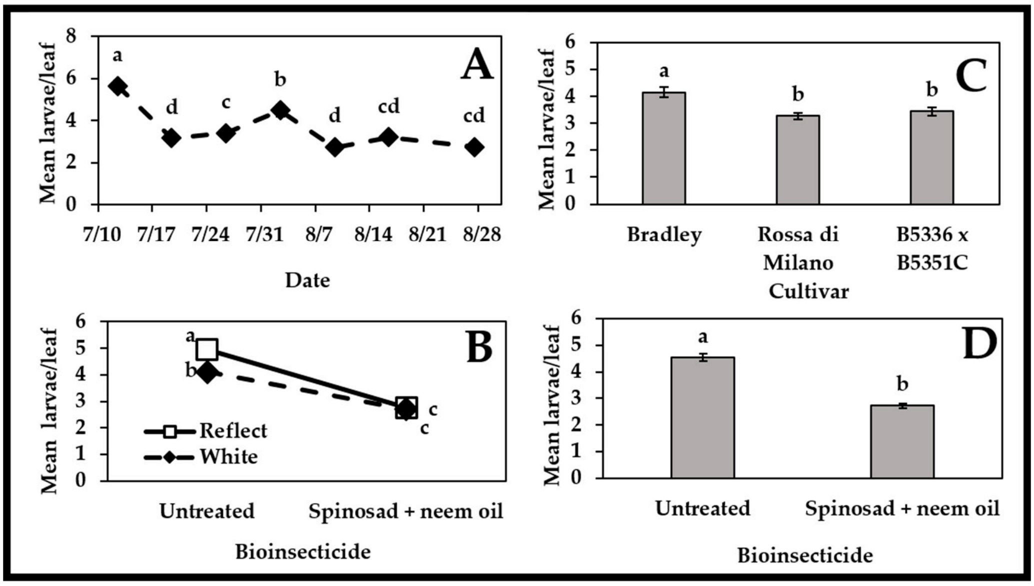

Figure S2. Significant lower order effects of interactions on larval thrips densities not included in the main text in the 2018 trial. Effects of date (A), the interaction of mulch by bioinsecticide (B), cultivar (D), and bioinsecticide (E) on mean (±SE) adult thrips densities in Geneva, New York, USA. Thrips densities are calculated as thrips/leaf. Means with asterisks (*) or different letters are significantly different at α = 0.05 using Tukey's LSD posthoc test. Letters are listed from top to bottom in the order of treatments in the legend.

2019

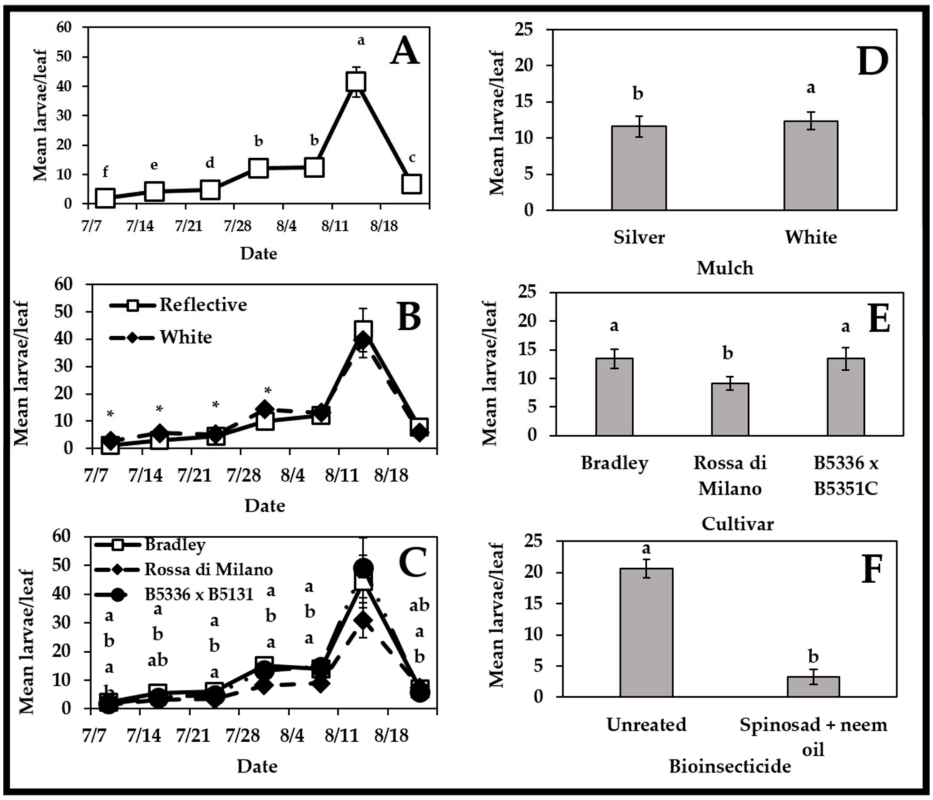

Figure S3. Significant lower order effects of interactions on larval thrips densities not included in the main text in the 2019 trial. Effects of date (A), the interactions of mulch by date (B) and cultivar by date (C), mulch (D), cultivar (E), and bioinsecticide (F) and on mean (±SE) adult thrips densities in Geneva, New York, USA. Thrips densities are calculated as thrips/leaf. Means with asterisks (*) or different letters are significantly different at α = 0.05 using Tukey's LSD posthoc test. Letters are listed from top to bottom in the order of treatments in the legend.

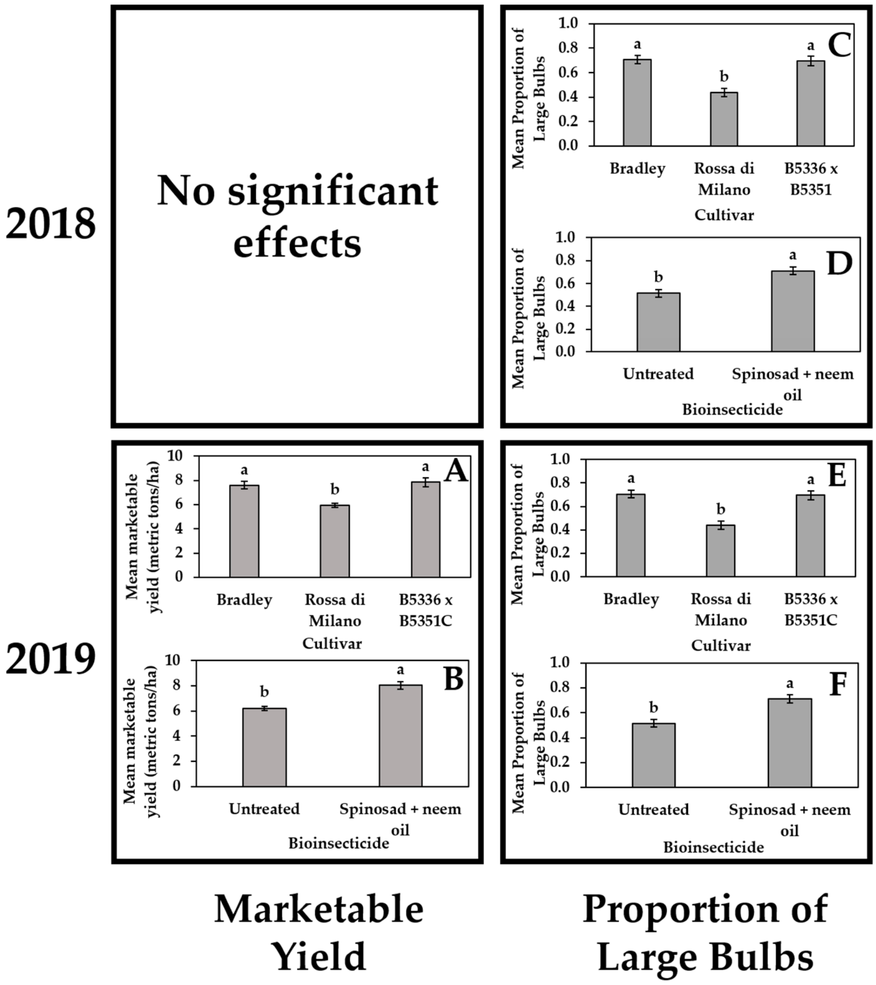

Figure S4. Significant lower order effects of interactions on bulb yield not included in the main text. Effects of cultivar (A, C, E) and bioinsecticide (B, D, F) on mean (±SE) marketable yield (metric tons/ha, A, B) and the mean (±SE) proportion of large bulbs (C-F) in 2018 (C, D) and 2019 (A, B, E, F) in Geneva, New York, USA. There were no significant effects on marketable yield not already included in the main text in the 2018 trial. Large bulbs are defined as having a diameter >7.3 cm. Means with different letters are significantly different at α = 0.05 using Tukey's LSD posthoc test.
